# A survey of practice patterns for real-time intrafractional motion-management in particle therapy

**DOI:** 10.1016/j.phro.2023.100439

**Published:** 2023-04-11

**Authors:** Ye Zhang, Petra Trnkova, Toshiyuki Toshito, Ben Heijmen, Christian Richter, Marianne Aznar, Francesca Albertini, Alexandra Bolsi, Juliane Daartz, Jenny Bertholet, Antje Knopf

**Affiliations:** a Center for Proton Therapy, Paul Scherrer Institute, Villigen, Switzerland; b Department of Radiation Oncology, Medical University of Vienna, Vienna, Austria; c Nagoya Proton Therapy Center, Nagoya City University West Medical Center, Nagoya, Japan; d Department of Radiotherapy, Erasmus University Medical Center (Erasmus MC), Rotterdam, the Netherlands; e OncoRay – National Center for Radiation Research in Oncology, Faculty of Medicine and University Hospital Carl Gustav Carus, Technische Universität Dresden, Helmholtz-Zentrum Dresden - Rossendorf, Dresden, Germany; f Faculty of Biology, Medicine and Health, Division of Cancer Sciences, University of Manchester, United Kingdom; g F. Burr Proton Therapy, Massachusetts General Hospital and Harvard Medical School, Boston, USA; h Division of Medical Radiation Physics and Department of Radiation Oncology, Inselspital, Bern University Hospital, Bern, Switzerland; i Institute for Medical Engineering and Medical Informatics, School of Life Science FHNW, Muttenz, Switzerland

**Keywords:** Particle/proton therapy, Intrafraction motion, Real-time respiratory motion management, Image-guided particle therapy, Rescanning

## Abstract

**Background and purpose:**

Organ motion compromises accurate particle therapy delivery. This study reports on the practice patterns for real-time intrafractional motion-management in particle therapy to evaluate current clinical practice and wishes and barriers to implementation.

**Materials and methods:**

An institutional questionnaire was distributed to particle therapy centres worldwide (7/2020–6/2021) asking which type(s) of real-time respiratory motion management (RRMM) methods were used, for which treatment sites, and what were the wishes and barriers to implementation. This was followed by a three-round DELPHI consensus analysis (10/2022) to define recommendations on required actions and future vision. With 70 responses from 17 countries, response rate was 100% for Europe (23/23 centres), 96% for Japan (22/23) and 53% for USA (20/38).

**Results:**

Of the 68 clinically operational centres, 85% used RRMM, with 41% using both rescanning and active methods. Sixty-four percent used active-RRMM for at least one treatment site, mostly with gating guided by an external marker. Forty-eight percent of active-RRMM users wished to expand or change their RRMM technique. The main barriers were technical limitations and limited resources. From the DELPHI analysis, optimisation of rescanning parameters, improvement of motion models, and pre-treatment 4D evaluation were unanimously considered clinically important future focus. 4D dose calculation was identified as the top requirement for future commercial treatment planning software.

**Conclusion:**

A majority of particle therapy centres have implemented RRMM. Still, further development and clinical integration were desired by most centres. Joint industry, clinical and research efforts are needed to translate innovation into efficient workflows for broad-scale implementation.

## 1 Introduction

An increasing number of cancer patients worldwide are treated with particle therapy (PT), and the number of new PT centres has grown remarkably over the last decade [1], [2], [3]. PT plans are characterised by high target dose conformity and increased healthy tissue sparing compared to photon external beam radiotherapy (EBRT). However, the quality of the delivered treatment can be substantially affected by inter- and intrafractional anatomical changes and organ motion [4], [5], [6], [7]. PT delivery is more sensitive to intrafraction motion than EBRT with the interplay effect being of particular concern [8], [9].

Many methods for real-time respiratory motion management (RRMM) have been proposed and applied to address intrafractional motion in PT. Simulations and experiments have shown that rescanning [10], [11], [12] mitigates the interplay effect, with the degree of efficiency depending on patient geometry, motion characteristics and the temporal dynamics of the delivery system [13]. However, rescanning cannot mitigate the reduction in dose conformity, due to respiratory motion. Therefore, for tumours with large motion, rescanning combined with active motion mitigation techniques such as beam gating [14], [15], [16], [17], [18], [19], [20] was suggested, whereby the use of a small gating window restricts the effective motion amplitude and reduces the number of rescans required to achieve acceptable dose homogeneity. Gating can be performed in breath-hold to minimise respiratory motion, depending on patient compliance [21], [22]. Gating in free-breathing comes at the cost of prolonged treatment times [15], increasing the risk of baseline shifts [23], [24] and patient fatigue. Moreover, it was proposed to steer the particle beam to synchronize the treatment delivery with tumour position, referred to as ‘tracking’ [25], [26], [27], [28].

RRMM effectiveness is patient-, equipment- and facility-specific, depending on clinical criteria and commercial or in-house-developed approaches [11], [13], [29]. Most RRMM studies for PT were conducted in a research context, based on in-house-developed software solutions, with limited commercial availability [30], [31], [32], [33], [34], [35], [36], [37], [38], [39]. While RRMM approaches like rescanning, gating or breath-hold are commercially available, comprehensive tools for realistic 4D evaluation of RRMM techniques are still limited. Although the challenges of treating moving targets were extensively studied, the actual status of clinical practice is largely unknown for PT centres. It is unclear how many PT institutions worldwide have RRMM capabilities and how many patients are treated with which type of technique. A recent study reported on the use of RRMM and adaptive radiotherapy in 200 EBRT centres [40], [41]. Although the challenges are similar in EBRT and PT, the clinical need is even higher in PT because of the dosimetric properties of the charged particle beams, and the technology (and commercial availability thereof) for motion monitoring and mitigation differs widely between EBRT and PT [4], [5], [42], [43].

In this study, we aim to determine the current status of the clinical use of RRMM for PT and to identify the barriers to clinical implementation through a global institutional survey. The survey was followed by a DELPHI consensus analysis among the authors to reach recommendations on the next steps from research, development and clinical implementation aspects.

## 2 Materials and methods

The Patterns Of Practice for Adaptive and Real-Time motion management in Particle Therapy (POP-ART PT) questionnaire was adapted from a survey to EBRT centres [40], [41]. The survey was addressed to clinical medical physicists and focused on current clinical practice at the institutional level, wishes for implementation and barriers to implementation. All 105 PT centres listed on the website of the Particle Therapy Co-Operative Group (PTCOG) as Facilities in Operation in 2020 [44] were invited to participate. The study was endorsed by the European SocieTy for Radiotherapy and Oncology (ESTRO), European Particle Therapy Network (EPTN) and PTCOG, and distributed through their mailing lists, social media, and personal contacts between July 2020 and June 2021.

This paper focuses on RRMM for intrafractional respiratory motion management. Plan adaptation to mitigate interfractional anatomical changes (APT) is addressed in a parallel paper [45]. Supplementary material (Suppl. A) contains the key terminology, questions, and summary. Responding centres (“responders” thereafter) were included in the analysis when they provided a complete response or only isolated questions were unanswered. Missing or uninterpretable answers were marked as ‘‘not specified”. Multiple answers from a single institution were merged and checked for consistency.

The questionnaire was completed by 70 PT centres from 17 countries worldwide, resulting in a response rate of 100% for Europe (23/23), 96% for Japan (22/23) and 53% for USA (20/38). Four centres from China and one centre from Thailand participated. Forty-three percent (30/70) of responders were academic clinical centre, and over 50% of responders have been in clinical operation for more than 5 years. Details on the responders can be found in Suppl. B. Two responders (3%, one in Europe and one in other regions) had not yet started clinical operation but filled the wish-list questions.

A three-round DELPHI consensus analysis [46] was performed with 11 experts (co-authors) to define recommendations for necessary developments and formulate a 10-year vision. The second and third rounds used controlled opinion feedback based on the previous round's responses. Experts responded based on their interpretation of survey results and personal opinions. Full consensus (FC) was reached when all experts agreed, and partial consensus (PC) when only one expert disagreed. When more than one expert disagreed, the statement was concluded as “no consensus” (NC). The details on the DELPHI process are provided in Suppl. C.

## 3 Results

**Fig. 1:**
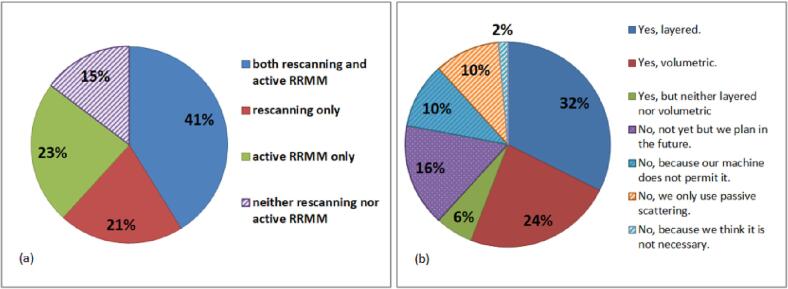
(a) Overview of RRMM implementation globally (N = 68) and (b) the situation of rescanning implementation (Q11: Are you applying re-scanning for any treatment site?).

Out of all clinically operational responders (‘clinical responders’ hereafter), 85% (58/68) had implemented at least one RRMM modality (either rescanning or active RRMM) (Fig. 1a). Here active RRMM are defined as techniques where either the patient actively complies (breath-hold, some ventilation techniques, gating with visual feedback) or treatment delivery is actively modified (gating or beam synchronization based on real-time motion monitoring). Fifteen percent (10/68) were not using any motion mitigation (one centre in Japan, three in USA, six in Europe). Sixty-two percent of clinical responders (42/68) used rescanning for at least one mobile treatment site, and 16% (11/68) planned to implement rescanning for a new treatment site in the next two years (Fig. 1b). Regionally (Suppl. B3), 59% (13/22), 70% (14/20) and 50% (11/22) of clinical responders in Europe, US and Japan used rescanning either alone or in combination with active RRMM. Seven clinical responders (three in Japan, three in Europe and one in US) cannot apply rescanning due to limitations of their equipment, and seven centres (six in Japan and one in Europe) had passive scattering systems for which rescanning is not applicable (or needed). Layered rescanning (delivering the divided spot weights multiple times for each energy layer before moving to the next energy layer) was more commonly used than volumetric rescanning (delivering the divided spot weights for the whole target volume multiple times). Four clinical responders used a hybrid rescanning mode. Large variations in number of rescans (2 to 6 times-per-field) were observed, in which 11 clinical responders applied patient-specific values, e.g. by considering max/min Monitor Unit per spot.

**Fig. 2:**
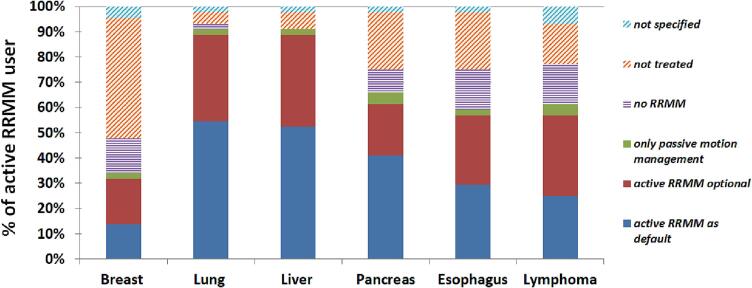
Percentage of active RRMM users (globally, N = 44) using different motion mitigations for various mobile treatment sites (Q13: Do you perform active RRMM for any treatment site in your particle facility?/Q14: Which treatment site are you treating with active RRMM?).

Globally, 65% of clinical responders (44/68) used active RRMM to treat at least one mobile treatment site (“active RRMM users” hereafter). Between 14 and 55% of clinical responders were applying active RRMM by default, depending on the treatment site (Fig. 2). The most common treatment sites treated with active RRMM were lung and liver, for which 89% (39/44) of active RRMM users used it either as default or optional. This was 61% (27/44) for pancreas, 57% (25/44) for oesophagus, 57% (25/44) for lymphoma and 32% (14/44) for breast. When RRMM was used optionally, the inclusion criteria depended on indications, centres and regions and included e.g. physician preference, motion amplitude from 2 to 10 mm (free-text responses).

**Fig. 3:**
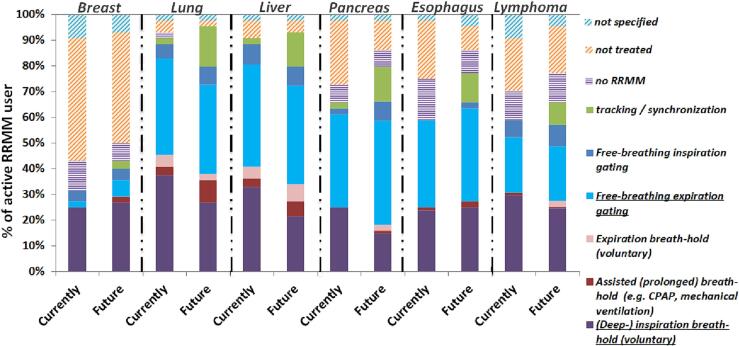
Status and wishes for the future regarding the RRMM technique for various mobile treatment sites, reported for active RRMM users only (globally, N = 44). Comparing two adjacent bars shows the difference between currently applied technique and what active RRMM users wish to implement for a particular treatment site. (Q17: Which active RRMM technique do you use currently/would like to use in the future?).

The most common RRMM techniques were voluntary deep inspiration breath-hold (29%) and free-breathing expiration gating (28%) (Fig. 3). Except for one user, active RRMM was applied for all liver and lung tumour treatments. Fifty percent (22/44) of active RRMM users stated their wish to change their current active RRMM routine for at least one treatment site. They wished to implement different active RRMM approaches for lung (43%), pancreas (34%), oesophagus (34%), liver (32%), lymphoma (30%) and breast (18%). Considering all treatment sites, the general trends were to increase active RRMM usage by 10%-point in average. Meanwhile, users wished to change their active RRMM technique to a more situation-specific selection in the future. Ten percent of active RRMM users wished to enable tracking/synchronization for all mobile treatment sites.

**Fig. 4:**
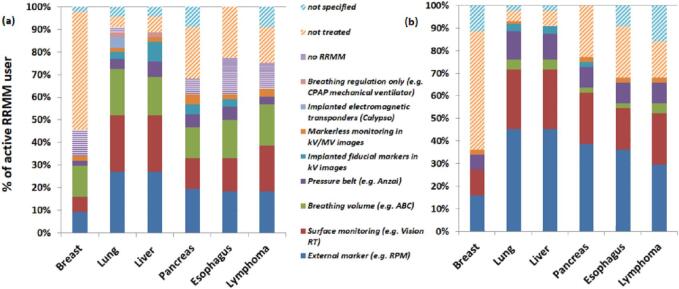
Usage of different motion monitoring signals for (a) guiding active RRMM delivery and (b) conducting 4DCT reconstruction. (Q20: Which motion monitoring signal are you using to guide the active RRMM procedure?/Q21: Which motion monitoring signal are you using for 4DCT sorting/reconstruction?).

The three most commonly used motion monitoring signals to guide RRMM were external marker (20%), surface monitoring (18%) and breathing volume (17%) (Fig. 4a). Motion monitoring was similar across all mobile treatment sites. The motion surrogates used for planning 4DCT sorting/reconstruction were generally different than those used to guide RRMM. An external marker was used by 35% of active RRMM users as surrogate for 4DCT reconstruction, while another 21% of users used surface imaging (Fig. 4b).

A dedicated coaching session was used by 55% of active RRMM users for at least one mobile tumour indication. For lung and liver, 55% (24/44) and 52% (23/44) of active RRMM users implemented coaching, mostly for 30 min. Moreover, 25% of active RRMM users had either audio or visual feedback during treatment delivery, with 18% (8/44) using both simultaneously (Fig. B5). For lung and liver, feedback was applied by more than 50% of active RRMM users. Less than 30% of active RRMM users acquired online imaging to verify the validity of the surrogate signal, including 16% (7/44) acquiring online verification imaging during beam-on for online review, while the other 14% (6/44) performed image acquisition for retrospective offline review. The majority of users doing image verification during beam-on were located in Japan and US.

**Fig. 5:**
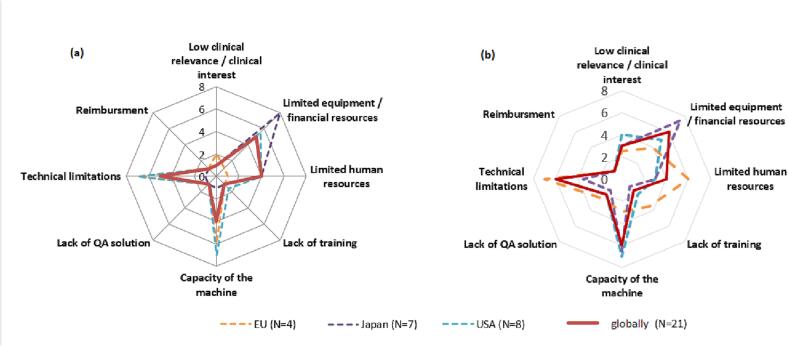
Scoring (median) of the main barriers to (a) extend or change the active RRMM technique for a site currently treated with RRMM and (b) to implement RRMM for a new treatment site.

Globally, 48% (21/44) of active RRMM users planned to expand the use of RRMM or change their technique for a treatment site already treated with active RRMM in the next two years (4/8 in Europe, 8/13 in US, 7/21 in Japan and 2/2 in other region). Priority was given to lung, liver and pancreas. All of them ranked the barriers with the top three challenges being technical limitations, limited equipment/financial resources and limited human resources (Fig. 5a). Reimbursement was lowly ranked both globally and regionally.

In total, 46% (32/70) of responders wished to implement active RRMM for a new treatment site. Of the current 44 active RRMM users, 30% (13/44) wished to implement RRMM for a new indication, with priority given to liver, lung and breast. For non-RRMM user (26/70), 73% (19/26) stated their wish to implement active RRMM, of which 42% (11/26) already had concrete plans for implementation in the next two years; 19% (5/26) had no wish to implement active RRMM for a new indication. The barrier for implementation of active RRMM for a new treatment site, were ranked by 63% (44/70) responders. The top three barriers were the same as for expanding/changing technique for currently treated indications (Fig. 5b). The regional ranking of barriers did not differ substantially from the global ranking.

**Fig. 6:**
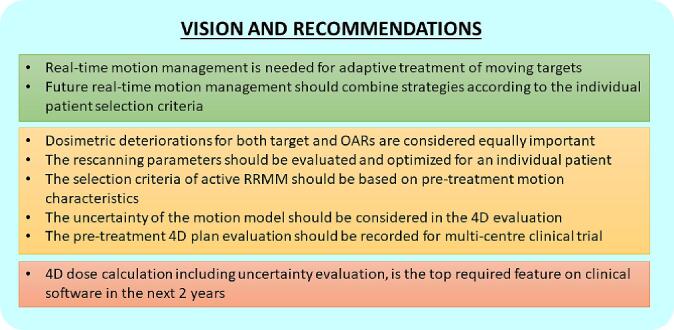
Summary of the most important conclusions from 3 round DELHI consensus analysis. Green box summarizes the vision, yellow boxes for general requirements and orange boxes for priorities. (For interpretation of the references to colour in this figure legend, the reader is referred to the web version of this article.)

The most important outcomes with full consensus (FC) from the DELPHI analysis were summarized in Fig. 6. More detailed results and other no consensus statements can be found in Suppl. C.

## 4 Discussion

This study provides a comprehensive picture of the clinical implementation of RRMM in 70 PT centers across 17 countries worldwide, including current status and future plans. While a majority of centers have adopted RRMM, many seek further improvements for better clinical integration. The study also outlines a 10-year vision for RRMM implementation, emphasizing the need for collaboration across industry, clinic, and research to translate innovation into clinical practice.

Globally, 85% of clinical responders used rescanning or active RRMM, with 41% using both, emphasizing the importance of motion management. Within two years, 78% of responders are expected to use rescanning, indicating that all centers capable of treating mobile tumors with rescanning will use it. However, the current heterogeneity of rescanning parameters necessitates consensus guidelines for effective implementation [9]. The DELPHI analysis achieved consensus on the need for standardized, individualized rescanning parameters based on pre-treatment 4D dose calculation and evaluation. The proton community must collaborate to standardize these parameters, considering equipment and model diversity. The 4D dose calculation should correctly estimate interplay effects by accounting for breathing cycle variations beyond standard 4DCT representations [47]. Dynamic beam models must also consider institutional scanning characteristics to properly individualize rescanning parameters [10–12,23,29]. Although this has been addressed by many studies [30], [31], [32], [33], [34], [35], [36], [37], [38], [39], not all features are clinically available [8]. According to the DELPHI results, the availability of 4D dose calculation and corresponding uncertainty evaluation in commercial software is a top priority to enable consideration of motion variability during 4D evaluation.

Sixty-four percent of clinical responders were active RRMM users, which is comparable to rescanning users (62%). More than 90% of the active RRMM users treated lung and liver tumours, with over 50% using it as default (Fig. 2). In Japan, 21/22 clinical responders were active RRMM users, with over 75% using it as default (Fig. B5), likely due to the availability of passive scattering systems readily capable of respiratory gating [19]. In contrast, EU and USA centres are more often equipped with active scanning beams, for which active RRMM is not as widely clinically available. When active RRMM was used optionally, inclusion criteria varied widely among indications and centres. The DELPHI analysis highlighted the need to choose the optimal RRMM technique based on pre-treatment motion characteristics but no consensus was reached on prioritizing a specific RRMM technique. Image-guidance, motion monitoring, and active compensation techniques were mentioned, but no clear prioritization emerged.

Besides compensating for motion (e.g. rescanning, gating and tracking), reducing motion (e.g. breath hold [22], [48], abdomen compression [49], [50] or patient selection [51]) can reduce the complexity of 4D PT delivery. The survey did not cover patient selection or abdominal compression, but 25–35% active RRMM users implemented various breath-hold techniques for different indications, which depend on the intra- and interfractional BH reproducibility [52], duration [53], and patient capability [48]. Reducing treatment time can be achieved by decreasing energy switching time (particularly for synchrotron-based machine) [54], and by increasing the beam current [55] with special plan optimization [56].

Breathing surrogates were the main methods for motion monitoring in active RRMM, similar to EBRT [40], but only 30% of users reviewed verification images online or offline, compared to 43–52% in EBRT [40]. The difference may be due to the delay in the implementing advanced online image guidance in PT [1]. Technically enabling such feature is necessary, including avoiding potential interlock triggers in the beam monitor system due to the online x-ray imaging dose detection. Dedicated coaching and online audio/visual feedback which is less technically demanding can improve the breathing reproducibility, were implemented by more than 50% of users. Nevertheless, breathing cycle irregularity and baseline tumour drift compromise the stability of the correlation, especially in the abdomen [57].

Slightly less than 50% of active RRMM users had plans to expand their current active RRMM technique to new indications (lung, liver and pancreas), the rest had no wish to extend due to limitations from both technical and human resource aspects. However, as automatic techniques are emerging [58], [59], we believe that human resource limitation will become less problematic in upcoming years. Unfortunately, 34% of responders did not provide a specific answer here, making the full picture on further active RRMM implementation incomplete.

The 64% of active RRMM users in the present study is similar to the 68% in the 200 EBRT centres [40]. In EBRT, breast was the main treatment site for RRMM but only 45% and 29% of responders were users of RRMM for lung and liver respectively, whereas these were the main treatment site in PT with over 90% of users for both. Several similarities between PT and EBRT are to be highlighted. For example, gating was the main RRMM method in both EBRT and PT with tracking being available only to 10% of EBRT responders and only one PT centre. Of note, 62% of POP-ART PT responders used rescanning, but passive mitigation was not explicitly surveyed in POP-ART RT. Rescanning questions were only included in a general way, without specific questions by treatment site. Retrospectively, given the heterogeneity of the results as discussed above, details by treatment site would have been of interest and should be included in future surveys.

The higher percentage of active RRMM users for lung and liver in PT centres compared to EBRT centres may be due to patient selection, where PT centres treat moving indications only when a suitable motion mitigation strategy is in place [9]. Most EBRT centres must treat lung and liver patients, whether active RRMM is available or not. There was a large interest in RRMM for lung treatments in both surveys indicating that RRMM is considered important for this indication whatever the modality. The similar barriers to implementation in EBRT or PT, despite the differences in technology, indicates that common effort could lead to solutions beneficial for both EBRT and PT [42], [43].

The PT community is well aware of the importance of RRMM, which has been addressed in recent clinical guidelines [8], [60], [61], [62] and in the annual 4D workshop [4], [5], [6], [7], [43] Results from POP-ART RT and PT surveys are in line with the recent reports of the 4D workshops [6], [7], regarding standardized guidelines to guarantee consistency between protocols applied at different facilities. From this study, we recommend focusing primarily on the development of consensus guidelines for rescanning parameters and inclusion criteria for active RRMM, while technical development and implementation should prioritize direct internal motion monitoring or frequent/practical verification of surrogate accuracy and beam synchronization.

In conclusion, RRMM with rescanning, active methods or both has been implemented by the majority of PT centres. Substantial interests were shown to implement more active RRMM, requiring joint efforts to address technical limitations and lack of resources. More research and development to translate 4D functions integrated in efficient workflows are needed to extend the use of RRMM clinically. This will require synergistic action from the industry, users and future users to translate ongoing research into clinical applications.

## Declaration of Competing Interest

The authors declare that they have no known competing financial interests or personal relationships that could have appeared to influence the work reported in this paper.
