# Supplementary material for: A survey of practice patterns for real-time intrafractional motion-management in particle therapy
Source: Phys Imaging Radiat Oncol. 2023 Apr 11;26:100439. doi: 10.1016/j.phro.2023.100439 (PMC10133874; doi:10.1016/j.phro.2023.100439)
Supplement: Supplementary Data 1 [file mmc1.docx]

# **Supplementary material A**

Full survey for the part I of the POP ART PT

## **Definition of the key terminology***

(*not provided when conducting the survey)

- Active RRMM – techniques for which the patient actively has to comply (breath-hold, some ventilation techniques, gating with visual feedback) or where treatment delivery is actively modified (gating or beam synchronization based on real-time motion monitoring)
- Tracking - synchronization of the beam delivery with the tumour position in real-time
- Passive mitigation – methods that passively account for potential motion by applying margins or rescanning, or abdominal compression.
- external markers – any non-invasively placed markers
- surface monitoring – monitoring of the patient surface using structured light patterns.
- breathing volume – air volume per breath (breath-hold)

## **Summary of the key questions in the survey**

(1) General questions (Q1-Q10) common to part I and II covered demographics, type of institution (academic, public and/or private), number of patients treated per year, facility size, vendor and if the centre was fully operational.

(2) Questions on rescanning (Q11-Q12) without specification of individual treatment site.

(3) Status of active RRMM (Q13-Q23), techniques where either the patient actively complies (breath-hold, some ventilation techniques, gating with visual feedback) or treatment delivery is actively modified (gating or beam synchronization based on real-time motion monitoring);  for which treatment site; what are the patient selection criteria; what are the workflow and technological approaches, regarding coaching, motion monitoring signal type, audio/visual feedback, as well as the online image verification for surrogate signal during beam-on.

The following active RRMM strategies were considered: Voluntary breath-hold (deep inspiration or expiration), Assisted breath-hold (e.g. CPAP, mechanical ventilation), Free-breathing gating (at inspiration or expiration phase), and Tracking, defined as continuously realigning the target and the beam (spatial beam tracking or synchronized dose delivery)

(4) Wish-list and barriers (Q24-30): Responders who have implemented RRMM were asked if and how they wished to increase the use of RRMM or to improve their workflow for treatment sites already treated with RRMM. All responders (users and non-users) were asked whether they wished to implement RRMM for a new treatment site. In both cases, responders scored potential barriers to implementation. Institutions that were not (yet) using RRMM were explicitly encouraged to fill the ‘‘wish-list and barriers” questions. Barriers were scored from 1 (least important) to 8 (most important). Empty scores, whenconsidered not relevant were assigned the lowest score. Median scores were compared both globally and regionally.

## **Main contents for the survey**

## General questions (same for part I and part II)

**Q1:** What is the name of your institution?

**Q2:** In which country are you situated?

**Q3:** Your institution is ... (please tick all that apply):

- Private
- Public
- Academic

**Q4:** For how many years has your particle centre been in clinical operation?

**Q5:** How many patients is your institution currently treating with particle therapy per year (approximately)?

**Q6:** How many patients does your institution plan to treat with particle therapy in full ramp-up per year (approximately)?

**Q7:** How many clinical treatment rooms does your particle therapy facility have (excluding eye-lines)?

**Q8:** How many clinical treatment rooms are currently in operation in your particle therapy facility (excluding eye-lines)?

**Q9:** Which treatment delivery machine do you have?

- Varian
- IBA PPlus
- IBA Pone
- Mevion
- Hitachi
- Other (please specify)

**Q10:** Please provide an email address in case we need to contact you for clarification (voluntary)

## Rescanning

**Q11:** Are you applying re-scanning for any treatment site?

- Yes, layered
- Yes, volumetric
- Yes, but neither layered nor volumetric
- No, not yet but we plan in the future
- No, because we think it is not necessary
- No, because our machine does not permit it
- No, we only use passive scattering
- No, because (please specify)

**Q12:** If you apply re-scanning, how many re-scans do you perform?

- 2-4
- 4-6
- >6
- N/A
- Varies depending on the patient / indication (please specify)

## Real-time respiratory motion management (RRMM)

**Q13:** Do you perform active RRMM for any treatment site in your particle facility?

- Yes, continue to site-specific questions
- No, skip to plans and wish list selection

## Current status: active RRMM

**Q14:** Which treatment site are you treating with active RRMM?

| Site | active rrmm as default | active rrmm optional | only passive** motion management | no motion management | N/a |
| --- | --- | --- | --- | --- | --- |
| breast | 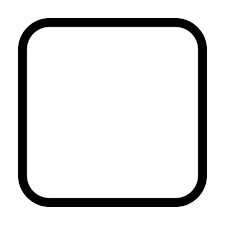 | 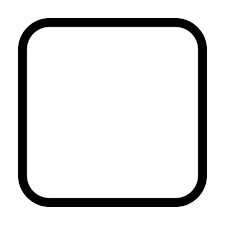 | 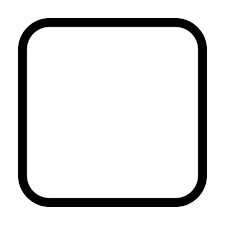 | 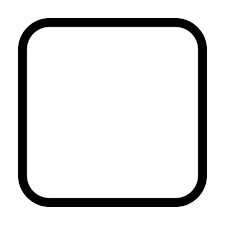 | 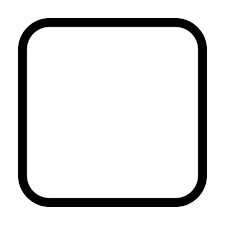 |
| lung | 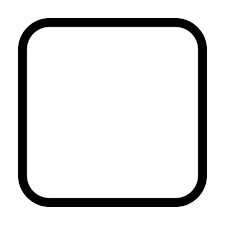 | 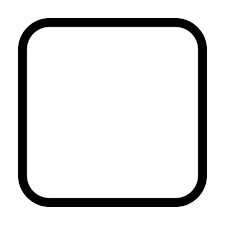 | 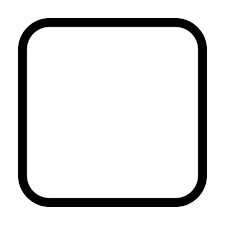 | 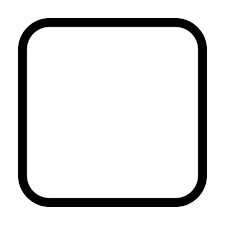 | 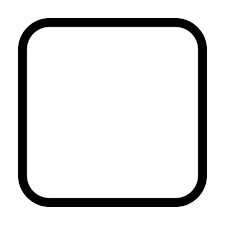 |
| Liver | 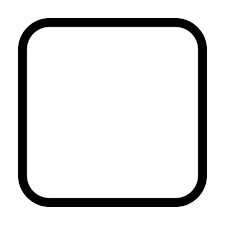 | 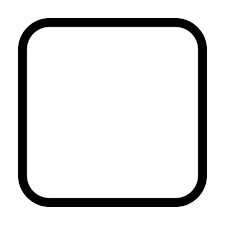 | 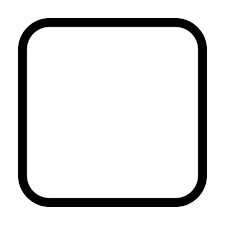 | 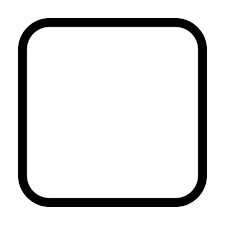 | 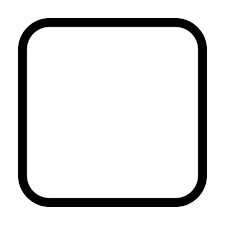 |
| Pancreas | 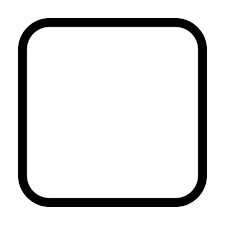 | 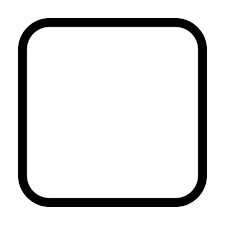 | 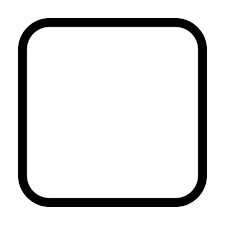 | 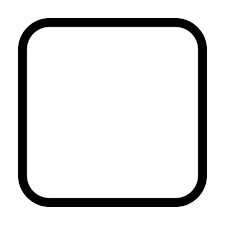 | 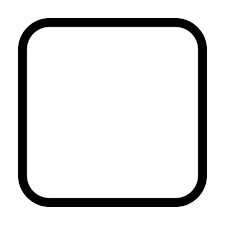 |
| esophagus | 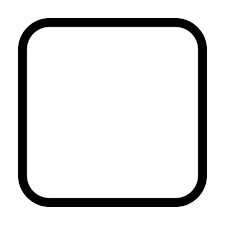 | 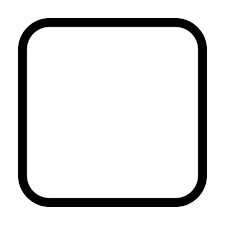 | 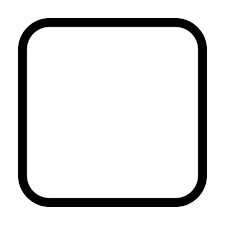 | 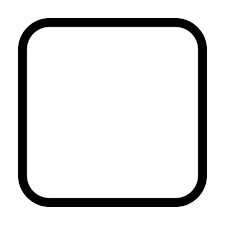 | 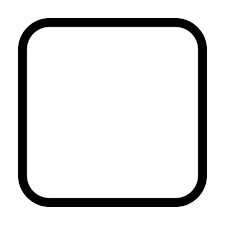 |
| Lymphoma | 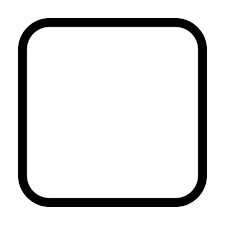 | 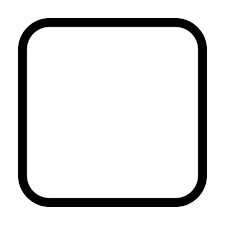 | 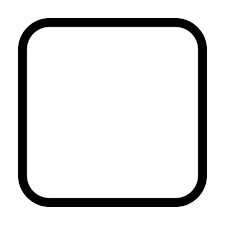 | 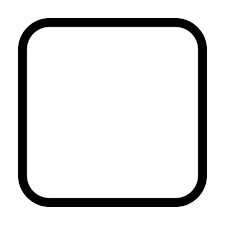 | 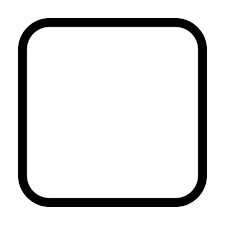 |
| OTHER* | 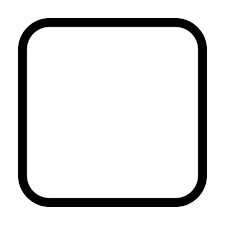 | 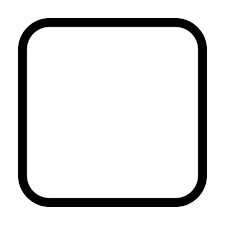 | 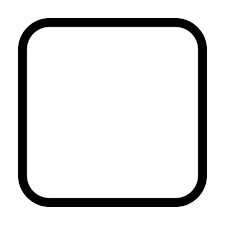 | 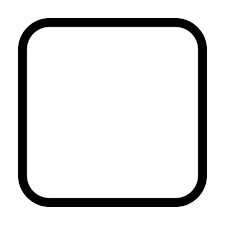 | 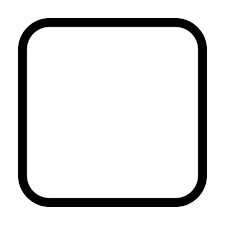 |

** Other (please specify)*

*** e.g. abdominal compression, rescanning*

**Q15:** How are you treating following treatment sites?

| Site | passive scattering | active scanning | not treated at our institute |
| --- | --- | --- | --- |
| breast | 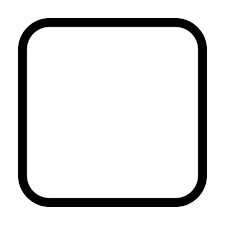 | 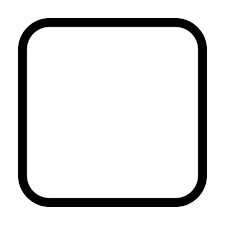 | 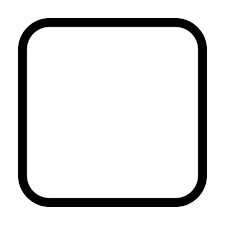 |
| lung | 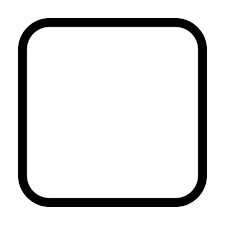 | 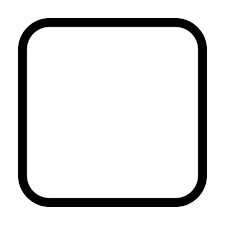 | 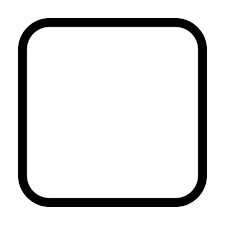 |
| Liver | 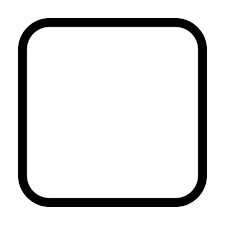 | 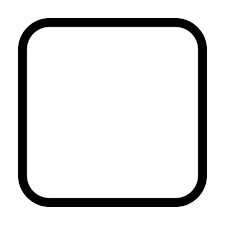 | 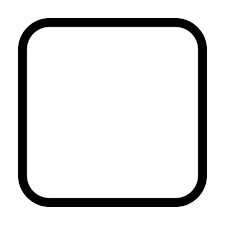 |
| Pancreas | 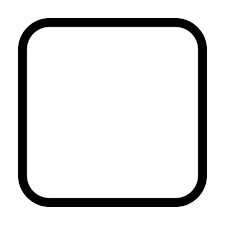 | 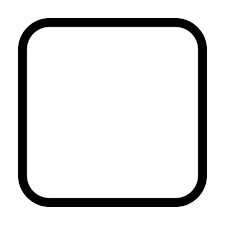 | 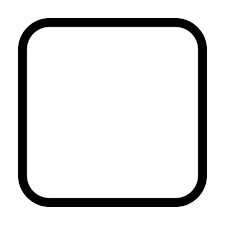 |
| esophagus | 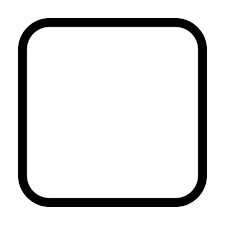 | 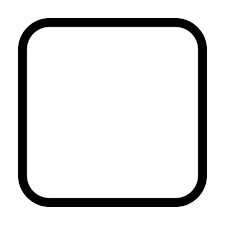 | 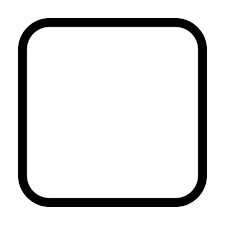 |
| Lymphoma | 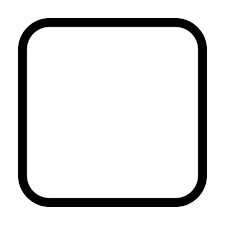 | 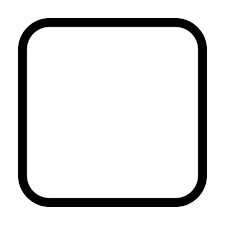 | 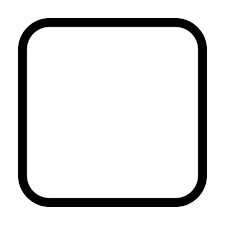 |

**Q16:** If you use “optional” active RRMM based on initial motion amplitude measure, specify your threshold motion amplitude in mm. If you use different criteria (e.g. physical performance, …) please specify under comments.

**Q17:** Which active RRMM technique do you use currently / would like to use in the future?

| Site | (Deep-) inspiration breathhold (voluntary) | Expiration breathhold (voluntary) | free-breathing inspiration gating | Assisted* (prolonged) breathhold | Tracking / synchro-nization | N/a |
| --- | --- | --- | --- | --- | --- | --- |
| breast Currently | 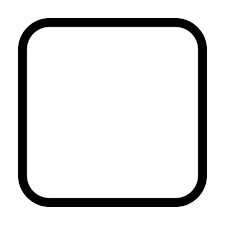 | 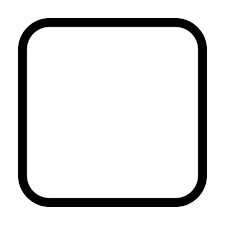 | 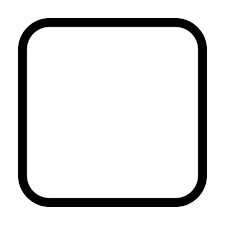 | 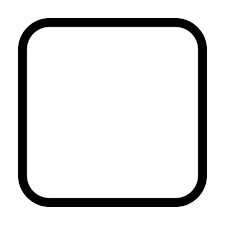 | 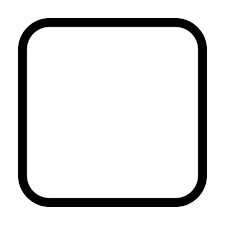 | 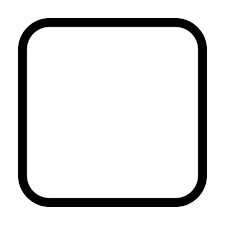 |
| Breast future | 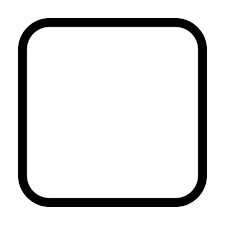 | 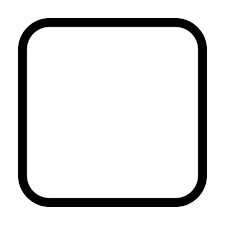 | 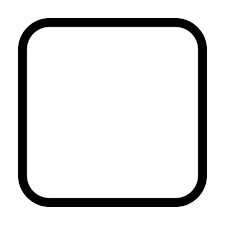 | 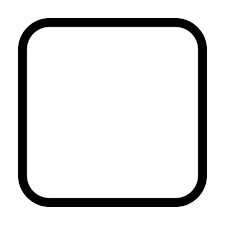 | 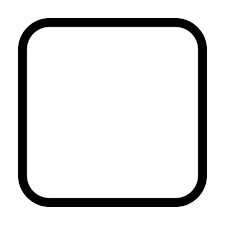 | 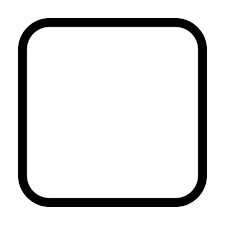 |
| lung Currently | 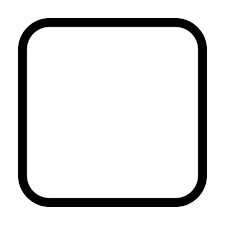 | 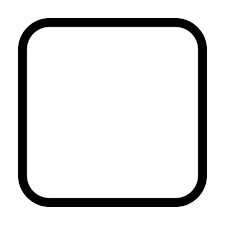 | 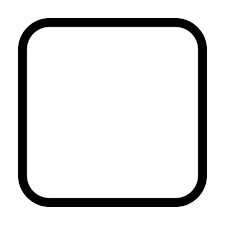 | 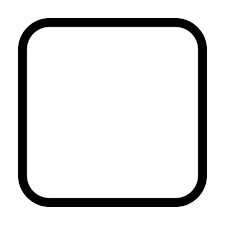 | 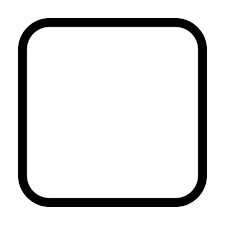 | 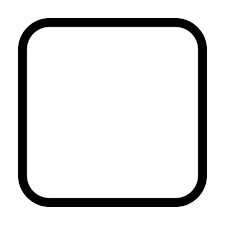 |
| lung future | 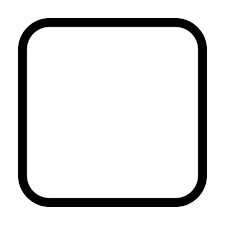 | 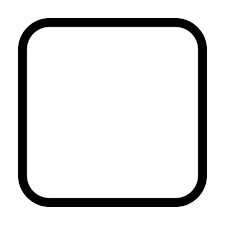 | 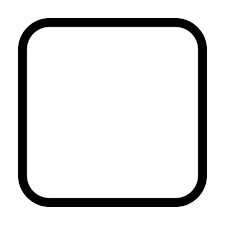 | 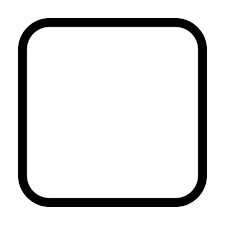 | 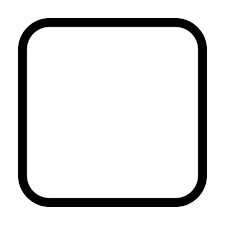 | 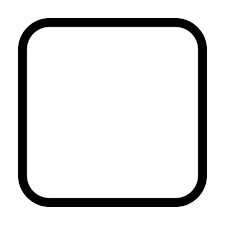 |
| Liver currently | 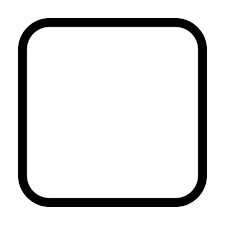 | 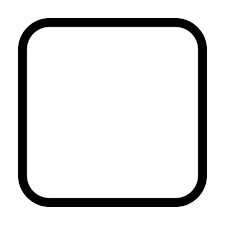 | 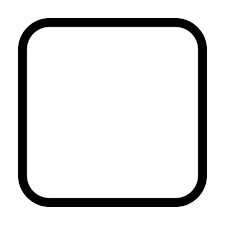 | 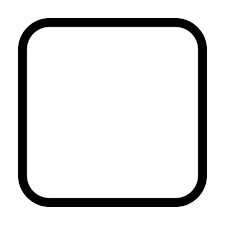 | 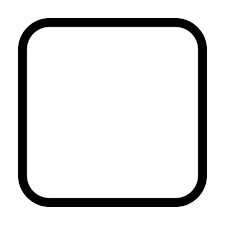 | 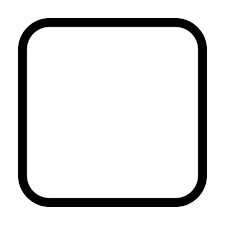 |
| liver future | 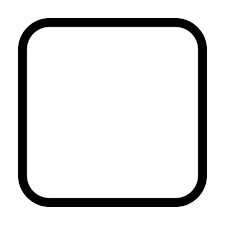 | 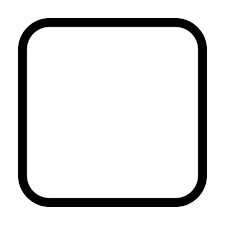 | 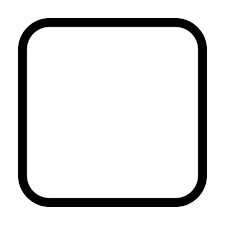 | 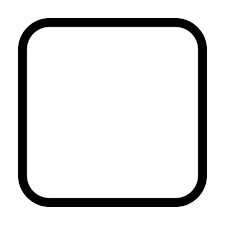 | 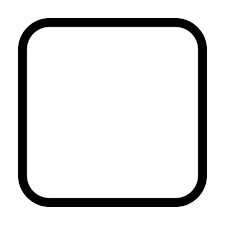 | 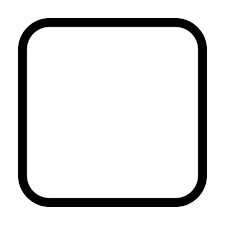 |
| Pancreas currently | 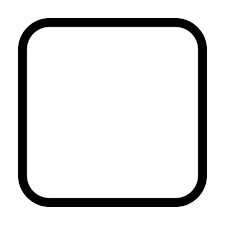 | 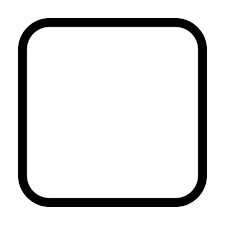 | 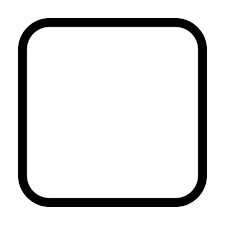 | 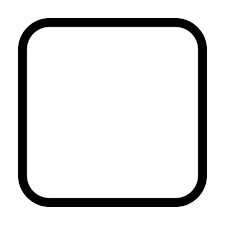 | 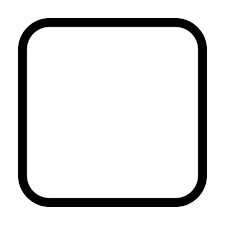 | 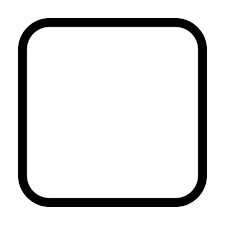 |
| pancreas future | 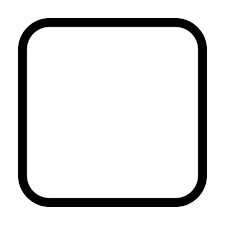 | 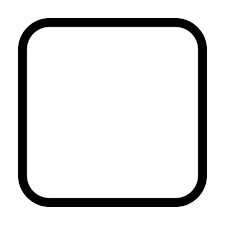 | 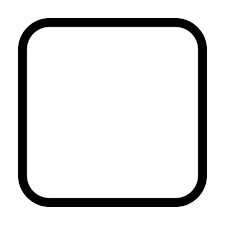 | 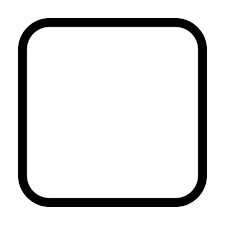 | 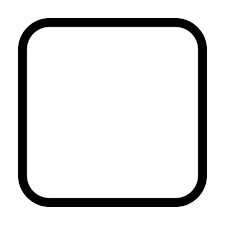 |  |
| esophagus currently |  |  |  |  |  |  |
| esophagus  future |  |  |  |  |  |  |
| Lymphoma  currently |  |  |  |  |  |  |
| lymphoma future |  |  |  |  |  |  |
| OTHER currently |  |  |  |  |  |  |
| other future |  |  |  |  |  |  |

** E.g. CPAP, mechanical ventilation*

**Q18:** What percentage of patients within each treatment site are treated with active RRMM currently?

| Site | < 25% | 25-50 % | 50-75 % | > 75 % | 100 % | N/a |
| --- | --- | --- | --- | --- | --- | --- |
| breast |  |  |  |  |  |  |
| lung |  |  |  |  |  |  |
| Liver |  |  |  |  |  |  |
| Pancreas |  |  |  |  |  |  |
| esophagus |  |  |  |  |  |  |
| Lymphoma |  |  |  |  |  |  |
| OTHER |  |  |  |  |  |  |

**Q19:** Do you have a separate training / coaching session for gating / breathhold?

| Site | Yes, please specify the time in min | no | N/A |
| --- | --- | --- | --- |
| breast |  |  |  |
| lung |  |  |  |
| Liver |  |  |  |
| Pancreas |  |  |  |
| esophagus |  |  |  |
| Lymphoma |  |  |  |
| OTher |  |  |  |

**Q20:** Which motion monitoring signal are you using to guide the active RRMM procedure? Here we mean the signal that is triggering the gating on / off or tracking feedback loop.

| Site | external marker (e.g. rpm) | surface monitoring (e.g. vision rt) | pressure belt (e.g. anzai) | implanted fidutial markers in kv images | implanted em transponders (e.g. calypso) | breathing regulation only (e.g. cpap, mech.vent.) | N/a |
| --- | --- | --- | --- | --- | --- | --- | --- |
| breast |  |  |  |  |  |  |  |
| lung |  |  |  |  |  |  |  |
| Liver |  |  |  |  |  |  |  |
| Pancreas |  |  |  |  |  |  |  |
| esophagus |  |  |  |  |  |  |  |
| Lymphoma |  |  |  |  |  |  |  |
| OTHER |  |  |  |  |  |  |  |

**Q21:** Which motion monitoring signal are you using for 4DCT sorting / reconstruction?

| Site | external marker (e.g. rpm) | surface monitoring (e.g. vision rt) | pressure belt (e.g. anzai) | implanted fidutial markers in kv images | implanted em transponders (e.g. calypso) | breathing regulation only (e.g. cpap, mech.vent.) | N/a |
| --- | --- | --- | --- | --- | --- | --- | --- |
| breast |  |  |  |  |  |  |  |
| lung |  |  |  |  |  |  |  |
| Liver |  |  |  |  |  |  |  |
| Pancreas |  |  |  |  |  |  |  |
| esophagus |  |  |  |  |  |  |  |
| Lymphoma |  |  |  |  |  |  |  |
| OTHER |  |  |  |  |  |  |  |

**Q22:** Do you use audio and/or visual feedback to the patient?

| Site | audio | visual | audio and visual | none |
| --- | --- | --- | --- | --- |
| breast |  |  |  |  |
| lung |  |  |  |  |
| Liver |  |  |  |  |
| Pancreas |  |  |  |  |
| esophagus |  |  |  |  |
| Lymphoma |  |  |  |  |
| Other |  |  |  |  |

**Q23:** If applicable, do you acquire online verification images (e.g. X-rays, surface images) during beam-on to verify the accuracy of a surrogate signal?

| Site | Yes, we look at them online | yes, but we review them offline | no | N/A |
| --- | --- | --- | --- | --- |
| breast |  |  |  |  |
| lung |  |  |  |  |
| Liver |  |  |  |  |
| Pancreas |  |  |  |  |
| esophagus |  |  |  |  |
| Lymphoma |  |  |  |  |
| Other |  |  |  |  |

## Plans and wishes: Intracfraction breathing motion management

**Q24:** Do you have plans to expand the use or change/improve your active RRMM technique for a treatment site currently treated with active RRMM in the next 2 years?

**Q25:** For which currently treated treatment site(s) do you wish to expand the use or change / improve your active RRMM technique in priority?

- Yes
- No

**Q25:** What are the main barriers / challenges to wider use of active RRMM in currently treated tumor sites? (rank in order of importance where 1 is the greatest challenge, leave the choices that are considered not relevant unmarked):

- Low clinical relevance/clinical interest
- Limited equipment/financial resources
- Limited human resources
- Lack of training
- Capacity of the machine
- Lack of QA solution
- Technical limitation (e.g. image quality, data connectivity, data flow, …)
- Reimbursement

**Q26:** Any other main barrier not specified above?

**Q27:** Do you have wishes to implement gating, active RRMM for a new treatment site?

- Yes, and we have plans to implement it in the next 2 years.
- Yes, but we have no clear implementation plan yet
- No

**Q28:** For which new treatment site(s) do you plan or would you like to implement active RRMM in priority?

**Q29:** What are the main barriers/challenges to implement active RRMM for a new indication? (rank in order of importance where 1 is the greatest challenge, leave the choices that are considered not relevant unmarked):

- Low clinical relevance/clinical interest
- Limited equipment/financial resources
- Limited human resources
- Lack of training
- Capacity of the machine
- Lack of QA solution
- Technical limitation (e.g. image quality, data connectivity, data flow, …)
- Reimbursement

**Q30:** Any other barrier not specified above?

# **Supplementary material B**

An overview of the general statistics of responding centres:

- Information on response rate: Table B1
- Academic status: Table B2
- Machine vendors: Table B3
- Years of operation: Figure B1
- Number of patients: Figure B2

Table B1: An overview of the numbers of responding centres and information on the clinical operation status.

| Region | Academic | Non-academic |
| --- | --- | --- |
| Europe | 10 | 13 |
| USA | 13 | 7 |
| Japan | 5 | 17 |
| Rest | 2 | 3 |
| **World** | **30** | **40** |

Table B2: An overview of the academic status of the responding particle therapy centres.

Table B3: An overview of the vendors in the responding particle therapy centres (N=70). *Combination=two different systems were used in a centre.

Figure B1: Overview of the years of experience of the responding particle therapy centres. The upper graph describes the experience of all the centres worldwide (N=70) and the lower plots describe the experience per region.

Figure B2: Overview of the number of treated patients per year at the operational responding particle therapy centres. The upper graph left describes the numbers of all the centres worldwide (N=64) and right the amount of the centres at a full patient capacity. The lower plots describe the patient numbers per region.

Figure B3 Overview of generally applied motion in different regions

Figure B4 Overview of the situation of rescanning implementation in different regions

(Q11: Are you applying re-scanning for any treatment site?)

Figure B5 Percentage of active RRMM users using different motion mitigations for varied mobile treatment sites regionally (Q13: Do you perform active RRMM for any treatment site in your particle facility? / Q14: Which treatment site are you treating with active RRMM? )

Figure B6 Usage of (a) online feedback and (b) image verification for various mobile treatment sites for verification of the surrogate signal during RRMM (Q22**:** Do you use audio and/or visual feedback to the patient? / Q23**:** If applicable, do you acquire online verification images (e.g. X-rays, surface images) during beam-on to verify the accuracy of a surrogate signal?)

# **Supplementary material C**

To reach a consensus on what are the most important developments, a survey was conducted among all co-authors of the manuscript, all experts in the field of adaptive radiotherapy using the DELPHI method.

A three-round questionnaire was used following the scheme in Figure C1. Second- and third-round questionnaires were adapted based on the answers from the previous round to provide controlled opinion feedback. The full consensus (FC), partial consensus (PC) or no consensus (NC) were reached when all experts agreed on an answer, only one expert had a different opinion or more than one expert had a different opinion, respectively.

The overview of the questions from the final round together with all the answers is presented in Table B2. Sometimes more than one answer to a question was possible. Not all the participants answered all the questions. Every participant had a possibility to comment on each question to more thoroughly elaborate on the answer.

Figure C1: structure of DELPHI consensus process based on 11 participants

Table C1: Overview of questions and answers of the third round of DELHI analysis. The numbers (x/n) in brackets indicate x= number of answers and n= number of participants. FC = full consensus, PC = partial consensus (only one expert disagree) and NC = no consensus (more than one experts disagree). The answers highlighted in bold correspond to FC.

| **#** | **Questions** | **Answers** | **Level of consensus** |
| --- | --- | --- | --- |
| **1** | **What is the primary need for RRMM in 4D PT treatment?** | margin reduction for normal tissue sparing | NC (0/11) |
|  |  | dose coverage/homogeneity for target dose conservation | NC (0/11) |
|  |  | **both equally important** | **FC(11/11)** |
| **2** | **What is the future real-time motion management approach** | Breath-hold | NC (0/11) |
|  |  | Rescanning | NC (0/11) |
|  |  | Gating | NC (0/11) |
|  |  | Tracking or motion-synchronized dose delivery | NC (0/11) |
|  |  | **Combined strategy** | **FC(11/11)** |
|  |  | No RRMM will be needed in future (e.g. as delivery will become fast by Flash technique) | NC (0/11) |
| **3** | **Should the criteria for RRMM selection be patient-oriented, treatment site oriented or institutional technique oriented?** | **patient** | **FC(11/11)** |
|  |  | Tumor site | NC (0/11) |
|  |  | Institutional technique | NC (0/11) |
| **4** | **Should the rescanning parameter be evaluated and optimized for the individual patient?** | **yes** | **FC(10/10)** |
|  |  | no | NC (0/10) |
| **5** | **What will be the absolute inclusion criteria for active RRMM** | Capability of breath-hold | NC (0/11) |
|  |  | **Motion characteristics at the pre-treatment phase (e.g. amplitude, frequency, reproducibility)** | **FC(11/11)** |
|  |  | Motion characteristics of individual fraction | NC (0/11) |
| **6** | **Should the below-associated uncertainty be considered in the 4D evaluation method?** | Uncertainty for patient model (e.g. interfractional anatomy changes) | NC(1/11) |
|  |  | **Uncertainty for motion model (e.g. motion variability, internal-external correlation)** | **FC(11/11)** |
|  |  | Uncertainty about dose summation using Deformable registration | NC (4/11) |
|  |  | Uncertainty for machine model (e.g. daily beam current fluctuation) | NC (0/11) |
|  |  | Uncertainty for dose calculation (e.g. Monte Carlo or analytical in 4D) | NC (0/11) |
| 7 | Do we need standardization across the institutes? | Yes, for the definition of rescanning parameters | NC (1/11) |
|  |  | Yes, for the definition of active RRMM inclusion criteria | NC (3/11) |
|  |  | Yes, considering the modality used for online motion monitor | NC (0/11) |
|  |  | Not yet, as first standardization in 4D treatment workflow must be reached | NC (9/11) |
|  |  | No because RMM is highly institution and patient-specific | NC (0/11) |
| **8** | **What should we record or verify in multi-center clinical trials?** | **Pre-treatment 4D plan evaluation** | **FC(11/11)** |
|  |  | Fractional 4D dose distribution | NC (9/11) |
|  |  | Daily image of the patient | NC (2/11) |
|  |  | Motion trace during each fractional dose delivery | NC (0/11) |
| 9 | is “fractional 4D dose distribution” necessary for a multi-centre clinical trial? (here means retrospective reconstructed dose distribution of each fraction, including the daily anatomy) | Yes | NC (7/11) |
|  |  | No | NC (4/11) |
| **10** | **Do you agree with the below features which the vendors need to implement in the next 2 years (no rank)** | **4D dose calculation and uncertainty evaluation** | **FC(11/11)** |
|  |  | Log file-based 4D dose reconstruction | NC (8/11) |
|  |  | Beam gating (including surface-guided and image-guided) | NC (9/11) |
|  |  | More efficient image guidance ((including low dose imaging, 4DCBCT, synthetic CT from CBCT) | NC (9/11) |
|  |  | others | NC (0/11) |
| 11 | Do you agree with the below topics still required research investigation in the next 5+ years (no rank) | provide evidence to demonstrate the clinical usefulness of RRMM | NC (8/11) |
|  |  | standardization of 4D workflow and establishing guidelines | NC (9/11) |
|  |  | Image-guided beam tracking and real-time plan optimization | NC (8/11) |
|  |  | Onboard MR-guided proton therapy | NC (9/11) |
|  |  | others | NC (0/11) |
